# A case presentation of acquired membranous ventricular septum aneurysm as a rare delayed complication of ventricular septal defect repair surgery: 10-year follow-up

**DOI:** 10.1186/s12872-023-03361-1

**Published:** 2023-07-12

**Authors:** Zhiyue Liu, Ting Liang, Siyu Jia, Wen Zhang, Yuan Feng, He Huang

**Affiliations:** 1 Department of Cardiology, West China Hospital of Sichuan University, Guoxue Xiang 37, Chengdu, 610041 China; 2 Department of Ultrasound, West China Second University Hospital, Sichuan University, Chengdu, 610000 China

**Keywords:** Acquired membranous ventricular septal aneurysm, Delayed complication, Ventricular septal defect repair surgery, Follow-up

## Abstract

**Background:**

Rare as membranous ventricular septal aneurysms (MVSA) is, the possibility that occurs after ventricular septum defect (VSD) repair surgery is even more uncommon.

**Presentation:**

A girl developed a MVSA 3 years after the VSD repair surgery at the age of 1 and increasing growth was noted during the follow-up. Aneurysm plication was carried done when she was 11 years old because it was observed to have a close relationship to the right coronary and obstructed the right ventricular outflow tract. Postoperative echocardiography follow-up revealed no abnormalities.

**Conclusion:**

Though the prognosis of most patients with VSD repaired surgery was good, there remains varieties type of complications despite surgical advances. Accurate and rapid diagnosis of acute and delayed complications is essential to improve prognosis. In this case, the aneurysm was diagnosed by multiple imaging modalities and the girl underwent successful surgery again which provides direction for awareness and knowledge of delayed complications of VSD repair.

**Supplementary Information:**

The online version contains supplementary material available at 10.1186/s12872-023-03361-1.

## Background

The membranous septum is a structure located in the midportion of the ventricular septum, situated just inferior to the aortic valve annulus. Membranous ventricular septal aneurysm (MVSA) is a rare finding, occurring in only 0.3% of cases and typically associated with congenital heart diseases [1, 2]. It is believed that most MVSAs are connected to spontaneous closure of ventricular septal defects (VSDs) [1]. However, MVSAs can be either congenital or acquired, and may also be idiopathic or develop after previous infection or trauma [3]. Postoperative MVSAs are relatively uncommon [4], which could be attributed to stitch pull-through resulting from VSD closure. Although many MVSAs are typically asymptomatic, they can also lead to significant complications such as thromboembolism, aortic valve leaflet prolapse, tricuspid regurgitation, right ventricular outflow tract (RVOT) obstruction, conduction abnormalities, or acute left-to-right shunting if the aneurysm ruptures due to disease progression [2]. Currently, there are no established management guidelines for treating an MVSAs in the absence of significant related complications or concomitant cardiac surgery. In this case, we present a child who developed an acquired MVSA after VSD closure surgery. The aneurysm was ultimately closed using continuous suture due to its proximity to the right coronary artery and RVOT obstruction.

## Case presentation

**Fig. 1:**
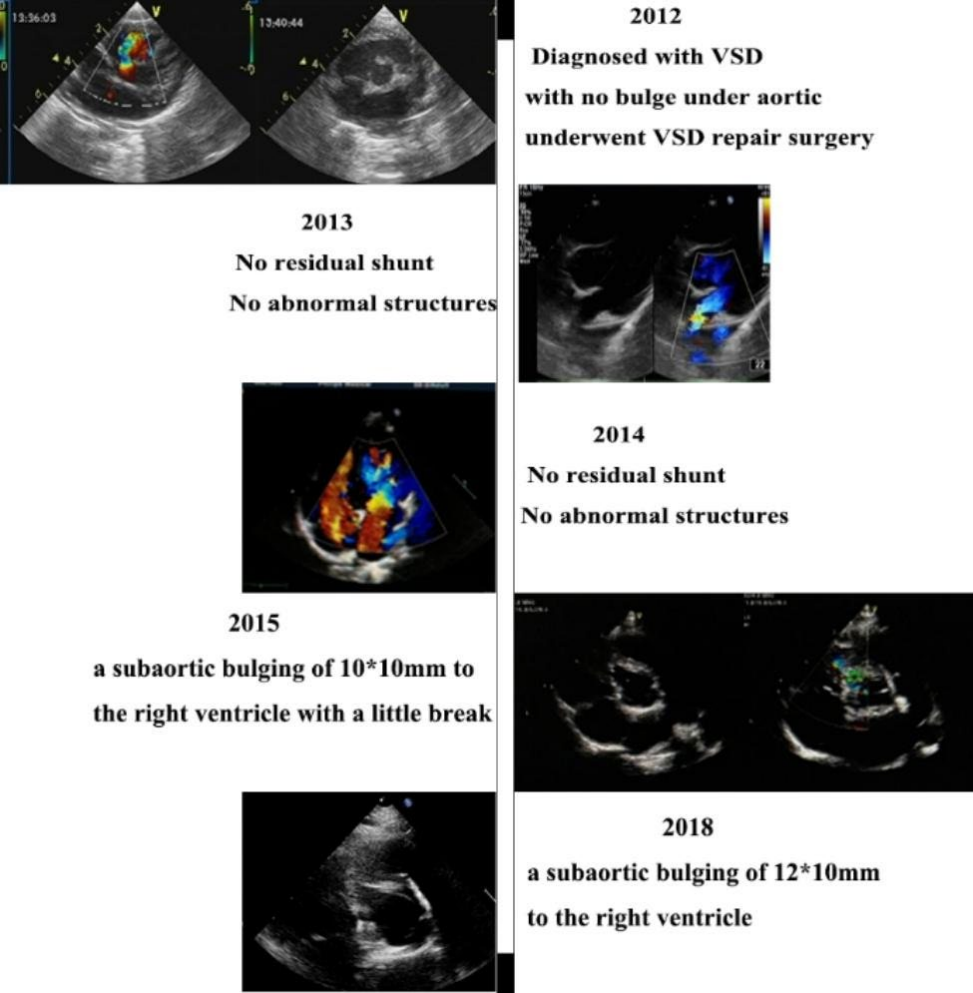
Case timeline details

**Fig. 2:**
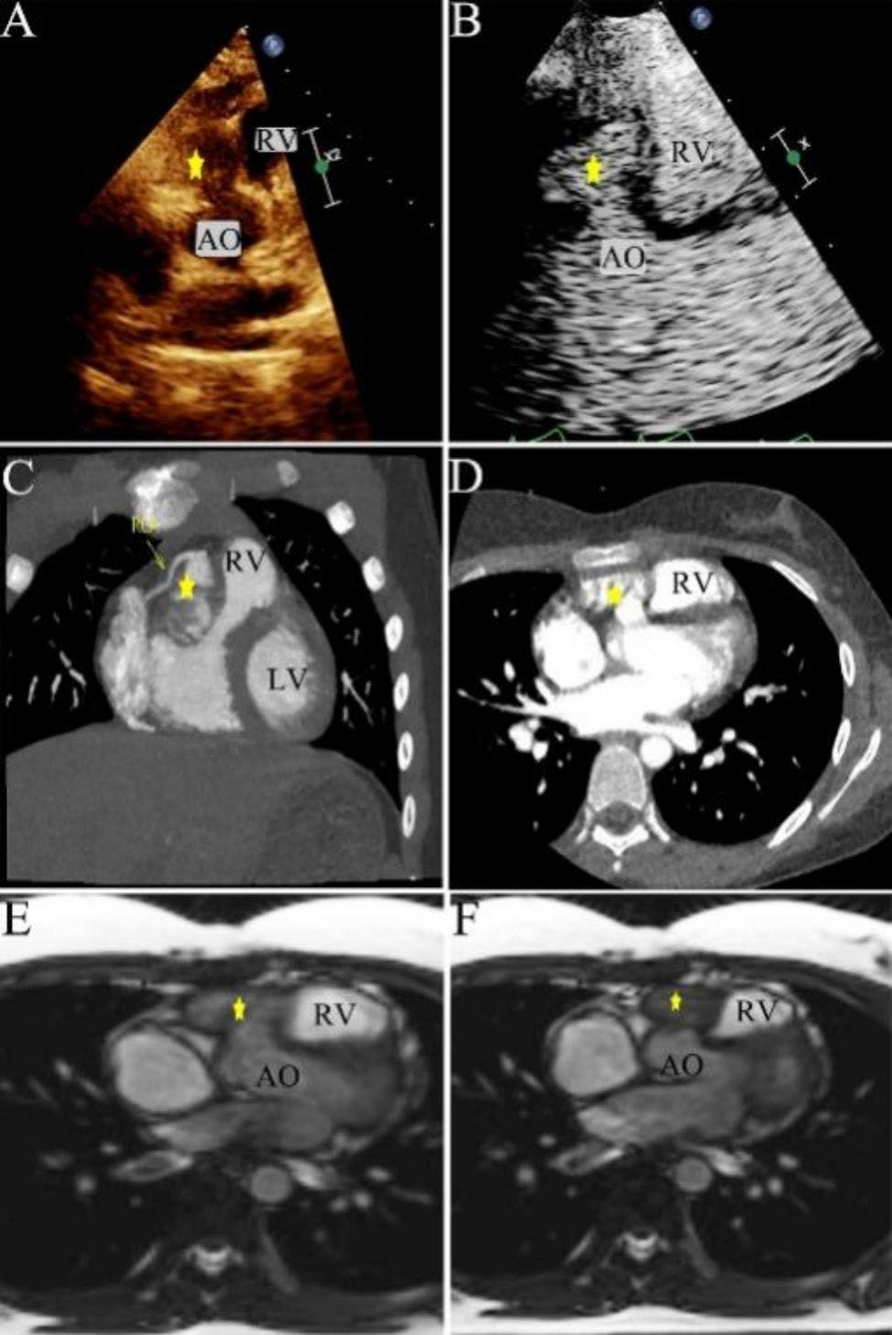
**A**. The echocardiography showed a bulge size of 21*13mm; **B**. The contrast echocardiography with sono vue showed a cystic structure opened under the aorta; **C**. The aneurysm had a close relationship to the right coronary. **D**. The aneurysm bulge to the right ventricle in enhanced-cardiac CT; **E-F**. Short-axis cine sequences of CMR showed a cystic shadow in front of the RV, **E**. at the end of diastole, **F**. at the end of systole; The cystic structure is designated by the yellow star. LV left ventricular; RV right ventricular; AO, aorta; RCA, right coronary artery

In 2012, a one-year-old girl underwent perimembranous VSD repair surgery which was measured 8 mm in size at local hospital. Between 2013 and 2014, no residual shunt or abnormal structures were detected. However, in 2015, transthoracic echocardiography (TTE) revealed the presence of a subaortic bulge measuring 10 × 10 mm with a small break into the right ventricle (RV). Conservative management was implemented with annual follow-up due to no obstruction and symptoms. Over time, the bulge progressively enlarged, as depicted in the case timeline (Fig. 1). In the latest TTE (at the age of 11), the bulge had grown even larger (21 × 13 mm, Fig. 2.A, Additional data online, Video S1) and the communication port showed bidirectional shunting. Accelerated flow was present in the RV outflow, with a peak systolic flow velocity of 2.6 m/s. Cardiac computed tomography (CT) (Fig. 2C-D, Additional data online, Video S3) and cardiac magnetic resonance imaging (CMR) (Fig. 2E-F, Additional data online, Video S4) revealed a cystic shadow measuring 40 × 20 mm in front of the RV. The aneurysm was found to be near the right coronary artery. During the 10-year follow-up, no positive symptoms or signs were observed, and myocardial markers remained within normal limits. No delay of growth and development was observed during the follow-up, as her height, weight, and head circumference were normal when compared to children of the same age.

To better understand the anatomical features and hemodynamics of the bulge, a contrast transthoracic echocardiography (TTE) was performed using *Sono vue (Bracco SpA, Milan, Italy)*. Following the injection of *Sono vue* contrast agent influx was observed filling a cystic structure (approximately 18 × 18 mm in size with a narrow neck measuring 8 mm) from the left ventricle (LV). No shunt was detected between the cystic structure and RV (Fig. 2.B, Additional data online, Video S2).

**Fig. 3:**
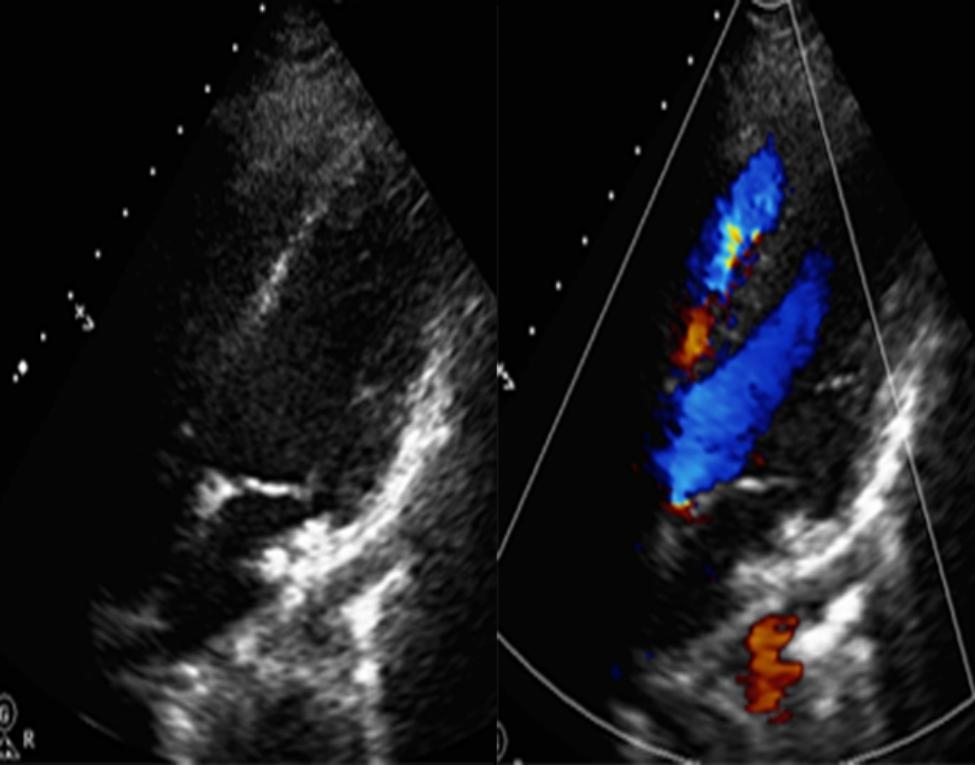
Plication postoperative TTE showed normal RV and LV morphology

Subsequently, surgical intervention was deemed necessary. The RV free wall was incised, revealing that the aneurysm opened into the LV outflow tract and was located beneath the junction of the non-coronary sinus and right coronary sinus (a common location for perimembranous VSDs). The aneurysm was enveloped by an outer layer of muscular tissue in the RV, and there was no communication between the RV and the right coronary artery. An acquired MVSA was diagnosed. Then, following the closure of the opening, the MVSA was plicated, sutured and reinforced with a Dacron patch through the aortotomy. Postoperative TTE showed normal RV and LV morphology (Fig. 3).

## Discussion

MVSA is characterized as an outpouching of the ventricular septum into the RV [5], which is often misdiagnosed as an aneurysm of the right sinus of Valsalva on TTE. This case is unusual in that the MVSA developed following VSD repair surgery. In children, LV outflow tract pseudoaneurysms have been reported after using biological tissue to close infundibular incisions [6]. The tissue immediately after surgical VSD closure is fragile and susceptible to damage, which can result in VSD patch dehiscence due to suture cutting through the tissue. In this case, the MVSA is a dilation of structurally weak regions of the VSD tissue that bulges into the RV as a result of LV pressure. The most probable cause of the MVSA formation may be the existence of a residual shunt following VSD repair, which is a common complication post-procedure and can be easily missed by echocardiography. Previous studies have shown that residual shunts < 4 mm in size can heal spontaneously. Roberts et al. [7] proposed that jet lesions are an important mechanism of self-healing in perimembranous VSDs, referring to reactive hyperplasia of the endocardium resulting from transseptal flow ejection, gradually covering the defect. Thus, it is reasonable to assume that the formation of MVSA may be a precursor to late spontaneous closure of residual shunts [2]. Additionally, the muscular tissue wrapped around the outer layer may be a result of intraoperative suturing, which over time covers the MVSA. The size of the defect, coexisting diseases, selection of patch material, and suturing technique are all factors that can impact the stability of the patch postoperatively, which would affect the probability of getting the aneurysm in future. Prior literature [8] has suggested that the patch size should correspond to the size of the defect base. Additionally, it is crucial to identify an appropriate incision during the procedure to expose the true border of the VSD. Unfortunately, the patient in our case underwent VSD repair surgery at another hospital 10 years ago. Although we have made efforts to trace it, we cannot fully describe the surgical details of VSD repair. However, we review the VSD repair surgery in our hospital 10 years ago, the materials included pericardium bobis, pericardium porch, autologous pericardium and Dacron patch. The stitching methods include continuous suture, interrupted suture and mattress suture.

VSD is the most common significant congenital abnormality, and surgical closure is the primary treatment, especially in children under one year old [9]. However, complications still occur. Rare complications of surgical VSD closure include LV pseudoaneurysm [10], aortic root pseudoaneurysm, subpulmonary pseudoaneurysm [11], and RVOT pseudoaneurysm [6]. These complications occur most frequently in the early postoperative period following the closure of a doubly committed VSD. Nandi et al. [4] reported a case of a five-year-old boy diagnosed with a moderate-sized (14 × 10 mm) MVSA after VSD closure surgery, which eventually ruptured in the RV, leading to severe tricuspid regurgitation with dilated right atrium and RV after two years. In our case, we observed a delayed complication of MVSA three years after surgery. Although no rupture occurred in this case, it grew excessively and caused RVOT obstruction, which eventually resulted in plication surgery again.

This case, with longest follow-up periods longer than any previously reported, is of special interest in light of recent reports suggesting that MVSA may be a mechanism for the spontaneous closure of small residual shunts. Surgical management of MVSAs should be performed when several conditions arise: (1) RVOT obstruction due to MVSA enlargement; (2) cardiogenic thrombosis results from hemodynamic changes in the aneurysm; (3) bacterial endocarditis requiring surgical treatment [12, 13]. Therefore, whether primary or secondary, if the risk of significant complications, including thromboembolism, aortic valve leaflet prolapses, tricuspid regurgitation, RVOT obstruction, conduction abnormalities, or acute left-to-right shunting secondary to aneurysmal rupture, are present, surgical or interventional treatment is still necessary.

## Electronic supplementary material

Below is the link to the electronic supplementary material.

Additional File Video 1: The latest TTE showed that the bulge size in left ventricular outflow tract was even larger (21*13mm).

Additional File Video 2: Contrast TTE showed a contrast agent influx could be seen filling a cystic structure (size of about 18*18 mm with a narrow neck of 8 mm) from the left ventricle (LV). No shunt was found between the cystic structure and RV.

Additional File Video 3: Cardiac computed tomography showed a cystic shadow of 40*20mm was seen in the front of RV, and the aneurysm was observed to have a close relationship to the right coronary.

Additional File Video 4: Cardiac morganic renounce computed tomography showed a cystic shadow of 40*20mm was seen in the left ventricular outflow tract which bulge to the right ventricular.

## Data Availability

The datasets used during the current study available are from the corresponding author on reasonable request.
